# Book Review: Games and Society in Europa

**DOI:** 10.3389/fpsyg.2020.596400

**Published:** 2020-11-05

**Authors:** Bartosz Prabucki

**Affiliations:** Institute for Sport and Education Development, Warszawa, Poland

**Keywords:** traditional sports, traditional games, cultural heritage, UNESCO, cultura 2000, tradition, modernity, education

## Summary of the Book

The reviewed book was published by the European Traditional Sports and Games Association (ETSGA/AEJeST) as the result of a fruitful cooperation and co-publication, achieved thanks to the contributions of many different institutions.

It consists of a preface, an introduction, two main parts and final reflections.

A short preface, written in three languages (English, French, and Spanish) introduces the reader into the subject of traditional sports and games (TSG)—the movement of their rediscovering, re-appreciation and promotion, starting from the 1980s and presenting its constant development until the special project, entitled “Juega con tu corazon, comparte tu cultura” (Play with your heart, share your culture), successfully carried out by the above-mentioned European Traditional Sports and Games Association with its European partners within the framework of the EU Culture 2000 Programme (2003-2006), which resulted in producing and interpreting data on traditional sports and games, played by the adults in 11 different, European countries and regions. The presented book was published by An introduction, written solely in Spanish, but with summaries in 7 different languages (among others in English, French, and Portuguese), deals with the thorough description of the above-mentioned project. This part contains a description of the background of this project, its main objectives, methodology, results, discussion, and conclusions.

The two main parts of this book deal with the detailed outcomes of the presented project.

The first part—entitled A Socio-Cultural Approach to European Traditional Sports and Games—contains 11 detailed articles, written in English, French, or Spanish (always with summaries in 7 languages), dealing with traditional sports and games from 11 European countries and regions—partners of the project.

The second part—Education in Traditional Sports and Games—consists of 2 articles in Spanish (with summaries), which concentrate on a very interesting example of educational activities with TSG, carried out in two Spanish regions—Cantabria and Catalonia.

The last part—Tradition and Modernity in Traditional Sports and Games—presents the final reflections on the international recognition for TSG from UNESCO, the Culture 2000 Project with its results and very important limitations, problems, and proposals for meeting future challenges in traditional sports movement in the twenty-first century. It is written in English, Spanish and French by the Scientific Director of the Culture 2000 Project—Pere Lavega.

## Evaluation

The presented book contains a rich and valuable material about traditional games and sports. The content of this work is the result of the above-mentioned, very important, European project, aimed at rediscovering, studying, analyzing, promoting, and finding practical applications for traditional sports and games—one of the most neglected, but at the same, most valuable parts of human cultural heritage. This is a common work of the distinguished scholars and practitioners, dedicated to this issue at the world's highest level. There are more than 300 games described or at least mentioned from different games' groups—ball games, bowl games, throwing, locomotion, shooting, or fighting games.

First of all, the reader can realize how important these games are, by getting to know with the international recognition for TSG as world cultural heritage from the most important, world-wide and European organizations, such as UNESCO or European Parliament, and by reading about a special organization—European Traditional Sports and Games Association—dedicated to revitalization, promotion, and practical application of traditional sports and games in Europe and all over the world.

Moreover, the reader can find here not only the detailed descriptions of numerous, European, traditional sports and games, but also their original photographs, tables and figures, indicating the interesting data, such as the number of traditional sports, still active on a given territory, their type, their features (called internal logic), and socio-cultural contexts in which they exist on a daily basis (external logic). The reader can find such games as: many varieties of Catalan bowl games, Portuguese Jogo do Pau, Polish Pierścieniówka (Ringnetball) and many others.

What is equally important, the reader can get to know with many practical applications of these games—they can be used in education, health-enhancing physical activity, social integration initiatives, tourism, recreation, education etc. The latter field is described in detail in the second, main part of this book, dealing with the very interesting examples of school and extra-curricular educational activities with traditional sports and games.

A limitation for the reach of this book is the fact that not all of its parts are written in English. Some of the articles are only in Spanish or French. To understand the entire content, the reader should know three languages. Fortunately, for every part, there are the before-mentioned summaries in 7 languages, helping the reader in general familiarization with the book's content.

## Discussion

The presented book deals with such an important and up-to-date issue that even today, after 14 years since its first publication, it is still very actual and universal. It contains a very interesting, theoretical assumptions, basing on Pierre Parlebas' motor praxeology and analysis of the research carried out within the framework of the above-mentioned project, giving the reader the necessary background to better understand the meaning of traditional sports and games.

In addition, it is enriched with many practical information, thanks to which everyone interested can get to know with numerous traditional games and sports—their descriptions, photos and practical applications. This is crucial as the reader can understand that these games and sports have a lot to offer, not only in theory, but also in practice.

Thanks to its well-structured way of narration, starting from the background of traditional sports movement, leading through the descriptions of many TSG from different parts of Europe and ending with indicating limitations, problems, and practical recommendations for the future, this book can serve as a starting point for everyone interested and for many world-wide, cultural, sport, social, and the other organizations to better grasp the significance of traditional sports and their importance for the future of our common world.

Specially significant is here introducing TSG to educational activities since, as Pierre de Coubertin said, the future of our civilization depends solely on the direction given to education.

## Author Contributions

The author confirms being the sole contributor of this work and has approved it for publication.

## Conflict of Interest

The author declares that the research was conducted in the absence of any commercial or financial relationships that could be construed as a potential conflict of interest.

